# The Usefulness of a Virtual Environment-Based Patient Setup Training System for Radiation Therapy

**DOI:** 10.3390/jimaging10080184

**Published:** 2024-07-30

**Authors:** Toshioh Fujibuchi, Kosuke Kaneko, Hiroyuki Arakawa, Yoshihiro Okada

**Affiliations:** 1 Department of Health Sciences, Faculty of Medical Sciences, Kyushu University, Fukuoka 812-8582, Japan; 2 Robert T. Huang Entrepreneurship Center, Kyushu University, Fukuoka 819-0395, Japan; 3 Data-Driven Innovation Initiative (DDIn2), Kyushu University, Fukuoka 819-0395, Japan

**Keywords:** virtual reality, patient setup, immobilization, radiation therapy, education

## Abstract

In radiation therapy, patient setup is important for improving treatment accuracy. The six-axis couch semi-automatically adjusts the patient’s position; however, adjusting the patient to twist is difficult. In this study, we developed and evaluated a virtual reality setup training tool for medical students to understand and improve their patient setup skills for radiation therapy. First, we set up a simulated patient in a virtual space to reproduce the radiation treatment room. A gyro sensor was attached to the patient phantom in real space, and the twist of the phantom was linked to the patient in the virtual space. Training was conducted for 24 students, and their operation records were analyzed and evaluated. The training’s efficacy was also evaluated through questionnaires provided at the end of the training. The total time required for patient setup tests before and after training decreased significantly from 331.9 s to 146.2 s. As a result of the questionnaire regarding the usability of training to the trainee, most were highly evaluated. We found that training significantly improved students’ understanding of the patient setup. With the proposed system, trainees can experience a simulated setup that can aid in deepening their understanding of radiation therapy treatments.

## 1. Introduction

Radiotherapy refers to therapy that uses ionizing radiation, generally as part of cancer treatment, to control or kill malignant cells. The procedure requires the administration of a sufficiently high dose to the clinical target volume of the tumor while ensuring that the radiation to the surrounding normal tissues is as low as possible. Thus, a high degree of local radiation control is required to irradiate the target accurately and with good reproducibility [1]. An important aspect of this controllability is the collation of the patient’s position every time regarding the treatment planning [2,3].

In recent years, the introduction of image-guided radiation therapy (IGRT) has greatly contributed to reductions in setup errors via position matching and image-based corrections [4,5]. In addition, couches with six freely moving axes have found use in radiotherapy units [6,7]. However, the twisting and distortion of the body axis cannot be corrected by adjusting the couch [8,9], which means that the treatment accuracy may be adversely affected by only moving the couch based on image guidance. If the patient remains tilted and organs are matched in IGRT, the couch may be tilted, which may cause the patient to become unstable. Therefore, to effectively utilize IGRT, we require a more accurate patient setup technique that allows medical staff to consistently align the skin marks with the isocentric lasers during patient setup to ensure that there are no changes in the patient’s position.

In recent years, virtual reality and augmented reality have been used for training in diverse fields [10], including radiography, as well as in the evaluation of radiotherapy plans [11,12,13,14,15,16,17]. Such training provides a hands-on experience without the need for expensive equipment or actual patients. Furthermore, the training can be carried out easily in almost any physical space. Here, we particularly note that radiation therapy equipment is expensive and also difficult to use for educational purposes. Thus, the teaching of radiation therapy technology to students aiming to become radiological technologists generally occurs in conjunction with clinical practice. At present, it is difficult to practice patient setup using actual radiation therapy equipment. In this regard, researchers have developed virtual-reality-based systems to address this issue [15,16,17,18,19,20,21,22,23,24,25,26,27,28]. Such a system allows students/trainees to practice setup operations using an actual controller and to superimpose computed tomography images of the patient and dose distribution in the treatment plan to deepen their understanding of radiotherapy. However, training based on patient positioning is difficult because, unlike in real-life scenarios, the positioning controls are usually keyboard-based, and students cannot make contact with the simulated patient or phantom, which makes the training difficult.

In addition, real patients are connected from head to foot, are heavy, and the isocenter is more than 1 m above the floor, making it difficult to twist the patient.

Against this background, our group developed a training system that facilitated patient setup by linking the virtual environment of the radiation therapy room with a real-world whole-body patient phantom. In this study, we used this system to train students in patient setup and validated the effectiveness of the training

## 2. Materials and Methods

### 2.1. Developed Virtual Reality Radiation Therapy Setup Training System

#### 2.1.1. Composition of the Radiation Therapy Setup Training System

In radiotherapy patient setup, skin marks on the patient’s body surface are used as external indicators of the target tumor. Lasers are emitted from the walls and ceiling of the treatment room to position the patient at the planned treatment location. In most facilities, two staff members are responsible for moving the treatment couch with the controller and for adjusting the patient’s torsional position via direct manipulation. Therefore, considering two staff members, we incorporated a setup procedure into the system in which one staff member was responsible for operating the couch in the virtual environment by means of a controller, and the other adjusted the torsion of the patient model in real space synchronized with the virtual space and aligned the laser with the skin marks of the patient in virtual space.

**Figure 1:**
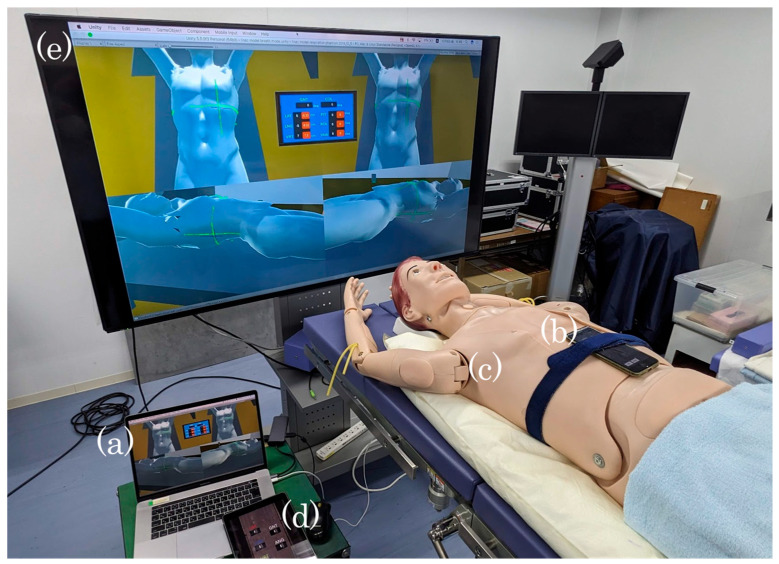
Our developed virtual reality-based patient setup training system. The system consists of server (**a**), gyroscopic sensors (**b**) with patient phantom (**c**), controller for operating the treatment couch (**d**), and large screen (**e**).

Figure 1 shows the configuration of the system we developed. As the training system, we developed a virtual space environment in the radiation therapy room using the game engine Unity 5.5.00 (Personal Edition, Unity Technologies, San Francisco, CA, USA). A 15-inch MacBook Pro (Apple Inc., Cupertino, CA, USA) was used to host the entire training system in the form of a server (Figure 1a). During patient setup training, an iPhone 7 (Apple Inc., Cupertino, CA, USA) (Figure 1b) was used to communicate the tilt information pertaining to a simulated patient’s whole-body phantom (Figure 1c), which was positioned on the bead cushion placed on a 110 cm high table. Detailed rotation adjustments were manually performed using a phantom with iPhone 7 (Apple Inc., USA) as the gyro-sensor, which was placed onto a couch positioned in real space. The students physically handled and adjusted the phantom. Such adjustments were made possible by synchronizing the rotation angles of all three axes detected by the gyro-sensors wrapped around the epigastric region of the whole-body phantom with the body position of the patient model in the virtual treatment room. In our approach, the translational movement of the patient model in the virtual treatment room was adjusted by moving the treatment couch by means of a tablet. An iPad Pro (9.7-inch, Apple Inc., USA) (Figure 1d) was used as a simulated controller to control the couch in virtual space. The MacBook Pro, iPhone 7, and iPad Pro were connected wirelessly via Wi-Fi. Information regarding the real-space tablet operation and the positional adjustment of the thoracic phantom was transferred in an interference-free manner to the virtual treatment room with the use of a network built using Node.js, which is an application run on a server in a MacBook Pro. As the output, we used a 50-inch LCD monitor to display the virtual environment for patient setup training (Figure 1e).

**Figure 2:**
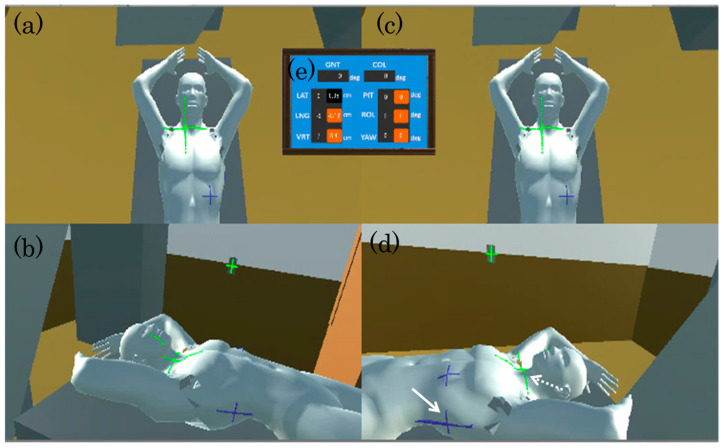
The screen of the radiation therapy room in virtual space. The left-screen view aids the personnel in charge of the patient’s torsion view from the ceiling (**a**) and the view from staff standing on the right side of the patient (**b**). The right-screen view guides the personnel controlling the treatment couch view from the ceiling (**c**) and the view from staff standing to the right side of the patient (**d**). The upper central panel in the screen shows the current couch position and gantry angle (**e**). The skin markers are placed on the patient model. Solid arrows indicate skin markers, and dashed arrows indicate isocentric lasers.

Radiation therapy equipment and a treatment couch, three isocenter-alignment lasers from the walls and ceiling, a whole-body model of the patient with both hands raised, and panels indicating the position of the couch and patient were constructed in the virtual space of the radiation therapy room. The patient was assumed to have lung cancer in the left lung field, and body surface markers were added to both sides of the body surface and the chest to indicate the tumor center. As shown in Figure 2, with this system, the radiation therapy room can be displayed on a large monitor, with the display divided into four views, two for the front of the simulated patient body (Figure 2a,c) and one each for the left and right sides (Figure 2b,d). In the procedure, the patient tilt and couch position are adjusted in real space, such that the markers on both sides and the chest side of the patient’s body are aligned with the lasers from the wall and ceiling. Skin markers and lasers on the patient model are shown in a virtual environment (Figure 2d). The skin markers are placed on the patient model. Solid arrows indicate skin markers (blue cross sign), and dashed arrows indicate isocentric lasers (green cross sign).

**Figure 3:**
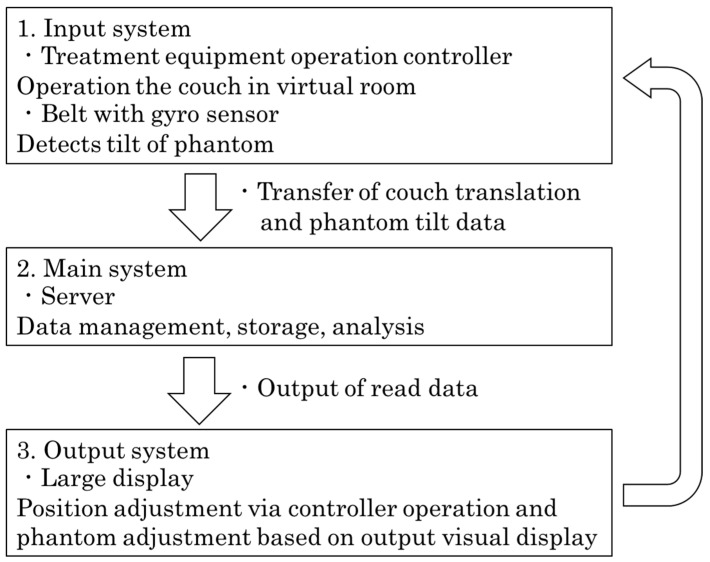
Flowchart of the radiotherapy setup training system. The virtual couch and patient are manipulated in the real world by means of the input system described in step 1. The virtual couch is linked with the phantom. In step 2, the operation data are managed, stored, and analyzed by the server of the main system. In step 3, this information is displayed on a large monitor, which is the output system.

Figure 3 shows the flowchart of the training system developed in this study. First, the data from the virtual couch and the input system (used to control the couch and phantom from real space) were transferred to the server of the main system. These data were managed, stored, and analyzed by the server and finally displayed on a large monitor, which was the output system. The operator inspected this screen and adjusted the position of the phantom and the couch.

During the procedure, the patient model in real space is first deliberately tilted at an angle and held by a cushion. A staff member on the left side of the patient then operates the couch using a controller and aligns the laser to the skin mark drawn on the left side of the patient.

#### 2.1.2. Tilt Detection of Simulated Patient and Control of Virtual Couch

**Figure 4:**
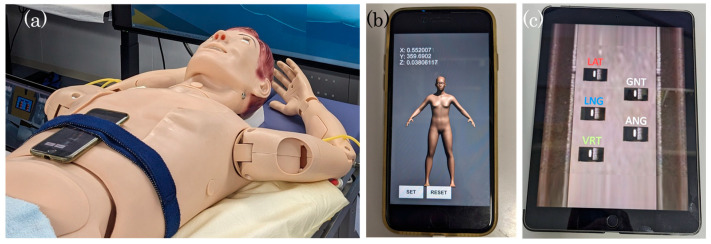
Real-space controller for a simulated patient and virtual radiation therapy couch. (**a**): iPhone fixed to the phantom by means of a belt. (**b**): iPhone and app for obtaining information on the tilt of the phantom. (**c**): iPad Pro and app for simulating the couch controller. The couch can be moved by pressing the buttons on the screen.

To adjust the tilt of a simulated patient in the virtual space, we used a whole-body phantom with an iPhone attached to the chest surface (Figure 4a). The iPhone was equipped with a Unity application designed for patient setup training, which used the iPhone’s built-in gyroscope to measure the angle of the phantom’s three axes at a rate of 30 times per second (Figure 4b). This angle information was transferred to the server (Figure 1a). Simultaneously, the detected angle and time were logged automatically. Here, we note that the gyroscope only detected the rotation angle, and therefore, the parallel movement of the phantom was not reflected. There is an idea to detect the translation of the phantom using an IMU (inertial measurement unit). However, in this training environment, the height of the couch in real space is fixed, and it cannot be raised, lowered, or slid using a controller, as in the case of a radiation therapy bed. This is in order to conduct training at a low cost. Therefore, since translation in the floor–ceiling direction is not possible in real space, and it would be strange to translate only in other directions, the translation of the couch can only be performed in the virtual space.

A bead cushion was placed under the phantom to stabilize and prevent it from moving after fine adjustment. The phantom and the installation cushions were set up on the platform with a height of 110 cm from the floor, thereby conforming to the height of the actual setup.

The therapeutic couch in the virtual space can be manipulated in real-time by a therapeutic controller reproduced on a 9.7-inch iPad Pro with a three-axis translation component (Figure 4c). The therapeutic couch in the virtual space is not linked to the actual couch. The tablet automatically records the pressed buttons and their times as a log, apart from the tilt information of the phantom.

**Figure 5:**
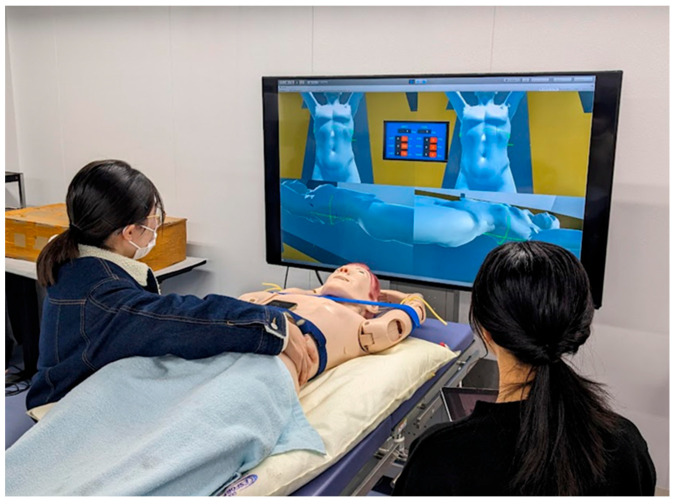
Screen partitioning during training to ensure that the operators cannot view each other’s screens. Student A stands to the right of the patient phantom and touches the phantom directly to adjust the tilt. Student B stands on the left side of the patient phantom and operates the bed in the virtual space with a tablet.

Student A stands to the right of the patient phantom and touches the phantom directly to adjust the tilt. Student B stands on the left side of the patient phantom and operates the bed in the virtual space with a tablet (Figure 5). Next, in the patient setup test, a partition is positioned such that students cannot view each other’s screen.

### 2.2. Flow of Patient Setup Training

This study was approved by The Ethics Committee of our institution. The patient setup training was conducted for 24 third-grade students undergoing a radiological technology course offered by the Department of Health Sciences. Six people attended each training course. These students had not completed their clinical training in radiotherapy. The students were first paired, and each pair was assigned to manipulate the phantom and the tablet during each training session. The flow and contents of VR patient setup training are shown in Table 1.

#### 2.2.1. Explanation of Setup Training (20 Min)

At first, we explained the purpose of the setup training, operation method, and patient setup for 20 min using a presentation slide.

#### 2.2.2. Pre-Training Setup Test (30 Min)

Next, the pre-training setup test was carried out. A setup test using a whole-body phantom that evaluated setup skills was administered to the students before and after training. The whole-body phantom was used as a patient role to evaluate the mastery of setup skills under the same conditions. The gyro sensor was able to evaluate the total time spent in the setup test, the number of operations of the simulated pendant, and the total time touched on each of the x, y, and z axes. Student A touches and positions the patient to adjust the inclination and twist. Student B operates a simulated pendant to move the couch in vertical and parallel directions. The students gained experience from Students A and B. Before starting the setup test, the phantom (linked to the simulated patient in the virtual space) was manually shifted in a random direction. This procedure was carried out to prevent the students from memorizing the moving direction and making adjustments without using the training system. After the completion of the test, the operators of the phantom and tablet exchanged roles, and training was carried out again in the same flow. After session completion, a questionnaire was administered to the trainees. During both the patient setup test and training, trainees were allowed to converse and advise each other.

When the setup test started, each trainee pair operated the tablet and phantom. Subsequently, both trainees reported setup completion to the administrator, and if the administrator acknowledged the correct completion of the setup, the training session for these was terminated. On the other hand, after the students’ declaration, if the administrator determined that the angle and couch position of the patient in the virtual space still exhibited deviation, the setup was continued; it was considered complete when the obtained values equaled the standard values.

**Figure 6:**
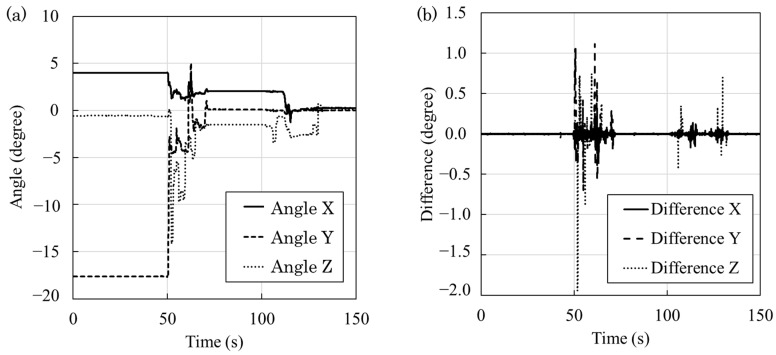
Angular log data of gyroscope. (**a**) Angular log of three axes. (**b**) Angular gradient log of three axes.

In the study, we analyzed the operation time and number of adjustments from the gyro sensor logs for each student pair undergoing the training. Examples of operation logs for the phantom are shown in Figure 6. In particular, Figure 6a shows the log of the angles of the three axes, while Figure 6b shows the log of the gradation of the angles of the three axes. In this figure, the vertical axis shows 0 when the student is not touching the phantom and making adjustments. The final slope of the *x*-, *y*-, and *z*-axes was set to 0, and the training was performed from the position where the slope was intentionally shifted.

#### 2.2.3. Patient Setup Training (80 Min)

In setup training, we carried out three intentions. One was to use a VR system, and while the students were simulating the patient, they practiced the setup and experienced interacting with a patient. The training time was about 40 min. Second, students watched a 360-degree video of the radiation therapy setup taken in advance using VR Google. The training time was about 20 min. Third, using the marked trunk phantom and laser marker device, we learned the relationship between the laser and the marker in real space. The training time was about 20 min.

#### 2.2.4. Post-Training Setup Test (30 Min)

After training, the same test as the pre-training test was performed.

### 2.3. Verification of the Effectiveness of the Training System

#### 2.3.1. Evaluation of Teaching Materials Based on the ARCS Model

In order to evaluate the entire setup of training, we gave students a questionnaire based on the ARCS model [29]. ARCS models consisted of 36 questions on the four items of attention, relevance, self-confidence, and satisfaction. The questions consisted of 12 questions about attention, 9 questions about relevance, 9 questions about self-confidence, and 6 questions about satisfaction.

#### 2.3.2. Contents of Questionnaire Administered after Training

The questionnaire administered to the trainees after training contained questions covering the following topics: effectiveness of training in conveying real-life situations, improvements in patient setup skills, confidence in performing patient setup, intuitiveness or lack of training tools, the appropriateness of training time, ease of manipulating the patient model, ease of couch manipulation, and ease of viewing the virtual environment radiation therapy room. The answers corresponding to these questions were graded on a 5-point scale, whereas a 4-point scale was used to grade their understanding of performing a patient setup.

Participants freely described their impressions of the training. We conducted an exploratory study to find the main points by conducting a multivariate analysis of the contents. The data were analyzed using KH Coder (Available for download at https://sourceforge.net/projects/khc/ (accessed on 28 July 2024)) for the qualitative text mining analysis to derive keywords related to the use of this teaching material from cluster thematic analysis, frequent word analysis and co-occurrence network analysis [30]. The KH Coder version 3 is a software program developed by K.Higuchi at Ritsumeikan University in Japan. This program consists of R programming language.

### 2.4. Verification of the Effectiveness of the Training System

**Figure 7:**
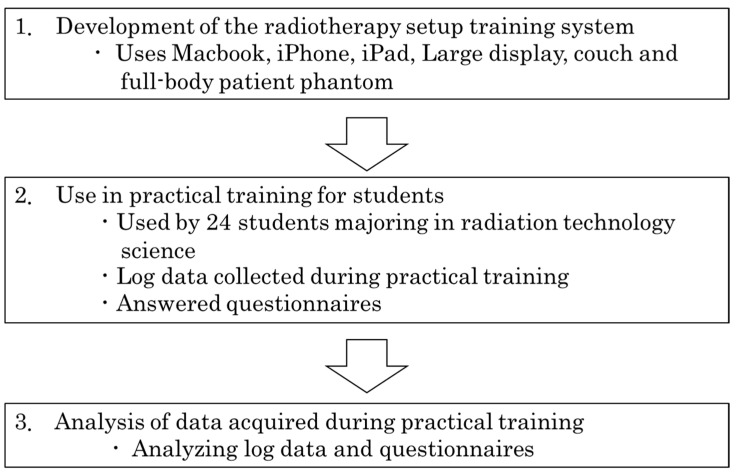
Flow of this research. Step 1 is the development of the system (details of the system are shown in Figure 3). Step 2 is the implementation of training (the training flow is shown in Table 1). Step 3 is the analysis of the data obtained during training.

The flow of this study is shown in Figure 7. This study was carried out in three steps. In step 1, the setup system shown in Section 2.1. was developed. In step 2, the training described in Section 2.2 was carried out. In step 3, the data obtained during the training, as shown in Section 2.3., were analyzed.

## 3. Results

### 3.1. Time to Touch and Move the Whole-Body Phantom

Table 2 shows the Results of the patient setup test before and whole-body training. For touching and moving the phantom, we noted that the measured value fluctuated within a range of ±0.004 (°) when the iPhone was stationary, and therefore, if the absolute value of the difference was greater than 0.004 (°), the phone was considered to have “moved,” and the amount of movement was defined as the error. The time decreased after training. The standard deviation was large due to the significant individual variability. For the number of times the buttons on the tablet were operated and the total setup time, we noted that after training, the number of operations was significantly reduced.

### 3.2. Evaluation of Practical Training Based on the ARCS Model

Table 3 shows the results of the mean value, standard deviation, average value of the total score of the four items of the ARCS model, standard deviation, ratio, and standard deviation of each question in the questionnaire based on the ARCS model. The total ratio of the three items, A, R, and S, was 80% or more, showing high values. However, the ratio of item C was lower than that of the other three items.

### 3.3. Questionnaire Results

The results of the questionnaire on the patient setup training system are listed in Table 4. This system was realistic; training improved patient setup techniques, training improved confidence in carrying out patient setup, the tool was intuitive and easy to use, the couch was easy to operate, the radiation therapy room in the virtual environment was easy to view, and more than 75% strongly agreed or agreed to show their consent. On the other hand, questions regarding whether training time was adequate and if patient models were easy to manipulate, the percentage of agreement was low.

In total, 87.5% of the students felt that the training assisted them in understanding how to perform the setup.

**Figure 8:**
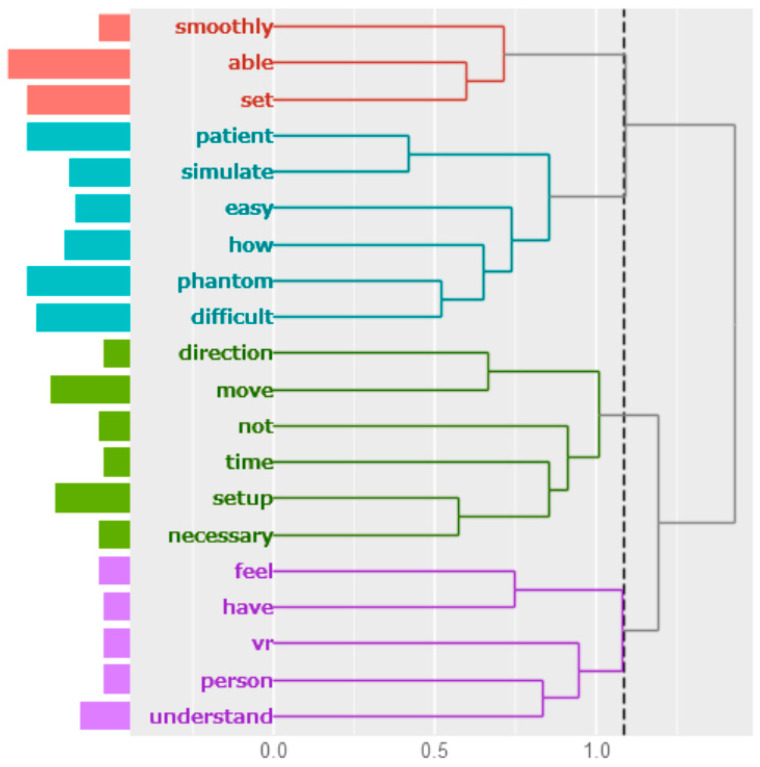
Cluster analysis about impressions of the VR patient setup training system.

In this study, 24 answers were collected as impressions of this training system. The results of the cluster analysis are shown in Figure 8. The free-answer text was divided into four clusters. Students were able to set up smoothly, had difficulty handling the phantom, felt the need for setup, and deepened their understanding when VRs were mentioned.

## 4. Discussion

Our training system shows that after training, (i) the phantom operation time reduced, (ii) the number of tablet operations reduced, and the (iii) total operation time shortened. These results can be explained as follows: firstly, inessential operations conducted in the wrong direction by the trainees reduced after training. During training, the patient position was displayed in segments on the screen and the administrator demonstrated the setup to the trainees, which helped them visualize the correct operation width and direction. Secondly, the trainees became familiar with the setup because of practice. Here, skill can be defined as the ability to perform a task based on knowledge as well as the ability to perform a task repeatedly. Thus, familiarity with an operation/task affords skill improvement. These findings indicate that the training provided a satisfactory understanding of the method of patient setup and aided in acquiring the relevant skills. However, since this training and testing was only conducted for a short period of time, the understanding and skills gained may be temporary. Thus, it may be necessary to reinforce the training after about a week to ensure that the procedure is fully understood and retained in the trainees’ memory.

Traditional training using only a phantom is shown in Table 1 3.C. Understanding the direction of phantom rotation using a phantom, and a laser maker device is essential, but this training is limited in its reproducibility, as it cannot reproduce the patient’s breathing or the raising and lowering of the couch.

As regards the items “I was able to imagine the actual scenario” and “I was able to operate the tool intuitively,” we note that the whole-body phantom used had a height similar to that of a real human body, thereby closely simulating an actual radiation treatment scenario. Furthermore, the tablet can be used as a pseudo pendant. In addition, the findings indicate that improvements in “setting-up” confidence, skill, and understanding occurred because the trainees were able to practically undergo a setup experience, which offered a vastly greater insight compared to simply viewing the actual setup.

The use of a phantom instead of an actual patient ensures that trainees can practice without time constraints as well as the stress experienced in the actual treatment environment. However, for an actual patient, it is necessary to consider the twisting of the body and the body parts that can be rotated without discomfiting the patient. The use of the phantom simplifies the adjustments that can be made to the patient. Therefore, training by attaching the iPhone to a student’s body can afford a better clinical experience and practice.

There are several limitations to this study. Only 24 students were included in this study. This is the number of students in one class who consented to the study. The sample size of the study was limited. Conducting the study with a larger number of students could collect more opinions, but this would take multiple years.

One example radiation therapy technician used this setup training system and was able to match the marker to the laser in a few touches. The visualization of the setup technique can be conducted by comparing the time of the procedure with the results of a group of medical staff with more clinical experience. This system may be effective for training not only students, but also new technologists assigned to the radiation therapy department. This is because the system is virtual and can be practiced repeatedly. A long-term study will be considered to observe the impact of using the system in an actual clinical setting.

## 5. Conclusions

In this study, we devised and evaluated the effectiveness of a patient setup training method for radiation therapy using a mixed-reality system. The average of the total time required for patient setup tests before and after training decreased significantly from 242.4 s to 150.2 s (*p* = 0.002). As a result of the questionnaire regarding the usability of the training to a trainee, most were highly evaluated. We found that the setup time was significantly reduced after trainees were trained with the proposed system. The system improved trainees’ understanding and skills in treatment setup by faithfully reproducing actual radiotherapy situations, which makes it difficult for trainees to train directly on the patient. The system is also effective for training because it can be used repeatedly, and the setup time and operation log information can be used for analysis of the technique.

## Figures and Tables

**Table 1:** Flow and contents of VR patient setup training.

1. Explanation of setup training (20 min);
2. Pre-training setup test (30 min);
Setup test using VR training system and whole-bodyphantom;
The phantom operation engineer role and sleeper operation engineer role are both performed while rotating.
3. Patient setup training;
A. Training using the VR system (40 min);
Students become simulated patients and use the VR training system;
Observe and comment on the operations of other students.
B. Watching a 360-degree video showing the flow of setup in the radiotherapy room (20 min);
C. Understanding the direction of phantom rotation using a phantom and a laser maker device (20 min).
4. Post-training setup test (30 min);
Same content as the pre-training setup test.

**Table 2:** Results of patient setup test before and after training (n = 24).

	Before Training	After Training	*p*-Value
Touch and move the chest phantom time (s)	29.3 ± 22.2	20.8 ± 18.5	0.050
Number of times the tablet buttons are pressed (times)	85.2 ± 45.5	58.5 ± 36.4	0.012
Total setup time (s)	242.4 ± 121.3	150.2 ± 85.0	0.002

**Table 3:** Result of ARCS model and questionnaire items for setup training.

Number	Item	Contents of Question	Mean Score	Standard Deviation
2	A	There was something interesting at the beginning of this educational content that got my attention.	4.02	0.85
8	A	This educational content was eye-catching.	4.23	0.68
11	A	The quality of this educational content helped to hold my attention.	4.32	0.56
12	A	This educational content was so abstract that it was hard to keep my attention on it.	4.07	0.93
15	A	This educational content looked dry and unappealing.	4.39	0.75
17	A	The way of presenting information about the setup training system helped to attract my attention.	4.02	0.63
20	A	This educational content had things that stimulated my curiosity.	4.02	0.59
22	A	The amount of repetition in this educational content caused me to get bored sometimes.	3.77	1.03
24	A	I learned some things that were surprising or unexpected through this educational content.	3.73	0.66
28	A	The variety of materials, such as slides, figures, videos, etc., used to explain this educational content using mixed reality helped keep my attention at the beginning of this practice.	3.95	0.71
29	A	The style of this educational content was boring.	4.07	0.93
31	A	There were so many explanations that it was irritating in this educational content.	4.11	0.87
6	R	It is clear to me how this educational content is related to things I already know.	4.23	0.80
9	R	There were stories, pictures, or examples that showed me how this educational content could be important to some people.	3.75	0.81
10	R	Completing this educational content successfully was important to me.	4.25	0.69
16	R	This educational content was relevant to my interests.	3.73	0.73
18	R	There were explanations or examples of how radiological technologists involved in radiotherapy use the knowledge in this educational content.	4.16	0.68
23	R	This educational content and style conveyed the impression that its content is worth knowing.	4.32	0.64
26	R	This educational content was not relevant to my needs because I already knew most of it.	4.14	0.85
30	R	I could relate this educational content to things I have seen, done, or thought about in my own life.	3.75	0.81
33	R	This educational content will be useful to me.	4.39	0.75
1	C	When I first looked at this educational content, I had the impression that it would be easy for me.	3.34	1.10
3	C	This educational content was more difficult to understand than I would have liked it to be.	3.75	1.08
4	C	After reading the introductory information, I felt confident that I knew what I was supposed to learn from this educational content.	4.05	0.78
7	C	This educational content had so much information that it was hard to pick out and remember the important points.	3.91	0.91
13	C	As I worked on this educational content, I was confident that I could learn the content of the setup.	4.45	0.55
19	C	This educational content used mixed reality was too difficult.	3.05	1.06
25	C	After working on this educational content for a while, I was confident that I would be able to pass a setup test.	3.52	0.73
34	C	I could not really understand most of this educational content.	4.23	0.71
35	C	The good organization of this educational content helped me be confident that I would learn setup.	4.09	0.56
5	S	Completing the exercises in this educational content gave me a satisfying feeling of accomplishment.	4.43	0.55
14	S	I enjoyed this educational content so much that I would like to know more about setup.	4.25	0.72
21	S	I really enjoyed studying this educational content.	3.98	0.59
27	S	The wording of feedback after the practice, or of other comments in this educational content, helped me feel rewarded for my effort.	3.75	0.75
32	S	It felt good to successfully complete this educational content.	4.16	0.57
36	S	It was a pleasure to work on such well-designed educational content.	4.23	0.57
A		Total	81.67	5.23
R		Total	82.22	3.62
C		Total	75.56	4.07
S		Total	80.00	2.64

**Table 4:** Results of patient setup training system questionnaire [%] (n = 24).

Statement	Strongly Agree	Agree	Unsure	Disagree	Strongly Disagree
This system was realistic	75.0	25.0	0.0	0	0
Training improved patient setup techniques	66.7	12.5	20.8	0	0
Training improved confidence in carrying out patient setup	62.5	12.5	25.0	0	0
The tool was intuitive and easy to use	50.0	41.7	8.3	0	0
Training time was adequate	20.8	33.3	37.5	8.3	0
Patient models were easy to manipulate	29.2	12.5	41.7	16.7	0
The couch was easy to operate	37.5	37.5	25.0	0	0
The radiation therapy room in the virtual environment was easy to view	50.0	25.0	25.0	0	0
	I understood the procedure before training	I understood the procedure after training	I understood it somewhat after training	I did not understand the procedure at all	
Understanding of performing a patient setup	29.2	58.3	12.5	0	

## Data Availability

The datasets presented in this article are not readily available because the data are part of an ongoing study. Requests to access the datasets should be directed to the corresponding author.
